# Exogenous addition of histidine reduces copper availability in the yeast *Saccharomyces cerevisiae*

**DOI:** 10.15698/mic2014.07.154

**Published:** 2014-07-07

**Authors:** Daisuke Watanabe, Rie Kikushima, Miho Aitoku, Akira Nishimura, Iwao Ohtsu, Ryo Nasuno, Hiroshi Takagi

**Affiliations:** 1 Graduate School of Biological Sciences, Nara Institute of Science and Technology, 8916-5 Takayama, Ikoma, Nara 630-0192, Japan.

**Keywords:** yeast Saccharomyces cerevisiae, basic amino acids, histidine cytotoxicity, copper transporter Ctr1, mitochondrial respiration

## Abstract

The basic amino acid histidine inhibited yeast cell growth more severely than lysine and arginine. Overexpression of *CTR1*, which encodes a high-affinity copper transporter on the plasma membrane, or addition of copper to the medium alleviated this cytotoxicity. However, the intracellular level of copper ions was not decreased in the presence of excess histidine. These results indicate that histidine cytotoxicity is associated with low copper availability inside cells, not with impaired copper uptake. Furthermore, histidine did not affect cell growth under limited respiration conditions, suggesting that histidine cytotoxicity is involved in deficiency of mitochondrial copper.

## INTRODUCTION

Free amino acids play pivotal roles as building blocks of proteins, as intermediates in metabolism, and also as regulators of a wide variety of cellular functions. In our previous studies, several amino acids including proline and arginine show cryoprotective activity in the budding yeast *Saccharomyces cerevisiae* 1,2,3. We recently showed that, under oxidative stress conditions, increased conversion of proline into arginine led to the flavoprotein Tah18-dependent synthesis of nitric oxide, which confers stress tolerance on *S. cerevisiae* cells 4,5,6. Although other charged amino acids, such as lysine and glutamate, also effectively enhance the freeze tolerance for yeast cells 1, the mechanism underlying the freezing stress tolerance by these amino acids remained unclear. In contrast, intracellular excessive levels of basic amino acids (lysine, arginine, and histidine) in mammals were reported to induce toxicity leading to gastrointestinal diseases, such as hepatomegaly and acute pancreatitis 7,8,9. Although pleiotropic disorders in lipid metabolism, protein synthesis, and mitochondrial functions have been observed in cells damaged by basic amino acids, their primary cellular effects are not fully understood.

Basic amino acids are incorporated via three similar permeases, Can1, Lyp1, and Alp1, which share 60-65% sequence identity with each other 10. Can1 was originally identified as an arginine permease 11, though it has been also reported to transport lysine, histidine, and ornithine with lower affinities 12. An ortholog of Can1 in *Candida albicans* actively transports lysine, arginine, and histidine 13. Lyp1 efficiently mediates only lysine transport 14,15. Overexpression of *ALP1* leads to specific uptake of arginine, although it is unclear whether this gene is expressed under physiological conditions 15. While these permeases transport amino acids with substrate preferences, the general amino-acid permease Gap1 is a transporter for all of 20 L-forms and also D-forms of the common α-amino acids, as well as other related compounds, such as citrulline, ornithine, γ-aminobutyric acid (GABA), and polyamines 16,17,18,19. Gap1 is most closely related to Hip1 in terms of amino acid sequence, although Hip1 seems to be a rather specific permease for histidine 15,20,21. Recent comprehensive studies revealed that single overexpression of these permeases decreases the growth rate 22,23, suggesting that *S. cerevisiae* can be utilized as a model to analyze the cytotoxicity caused by excess basic amino acids.

Among basic amino acids, histidine is especially related to copper transport 24. Since the discovery of copper(II)-bis(L-histidinato) complex in human blood 25, extensive research has been performed to determine its physiological roles. Consequently, histidine was found to facilitate copper uptake in hepatic, placental, and brain cells 26,27,28 by removing copper from albumin, which physically inhibits incorporation of copper ions 29. The copper(II)-bis(L-histidinato) complex has thus been applied for the treatment of Menkes disease and hypertrophic cardioencephalomyopathy, both of which are closely associated with copper deficiency 30,31. In *S. cerevisiae*, copper uptake is mediated by the high-affinity transporters Ctr1 and Ctr3 and a ferric/cupric reductase Fre1, which oxidizes copper(II) into usable copper(I) ions in advance of their uptake 32,33,34. To maintain copper homeostasis, the *CTR1*, *CTR3*, and *FRE1* genes are upregulated or downregulated under copper starvation or excess copper conditions, respectively, via the action of the copper-sensing transcription factor Mac1 35,36. Intriguingly, the mutations in the histidine biosynthetic genes of *S. cerevisiae* increase sensitivity to the excess amounts of copper, which is suppressed by addition of histidine 37. This finding supports the idea that histidine might directly interact with copper ions in yeast cells to alleviate the copper toxicity.

To explore novel roles of free amino acids, we analyzed here the cytotoxicity caused by exogenous addition of excess basic amino acids in *S. cerevisiae*.

## RESULTS AND DISCUSSION

**Figure 1:**
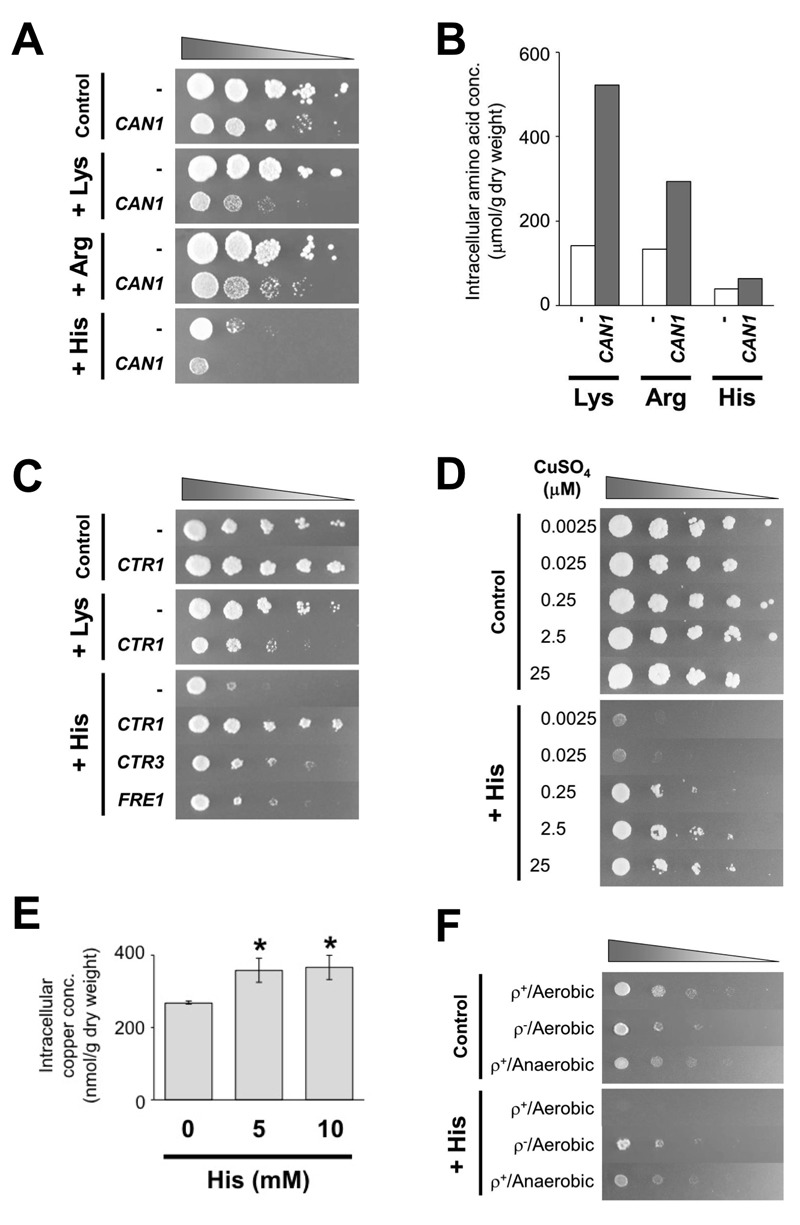
FIGURE 1: The effect of excess histidine on yeast cell growth and copper uptake. **(A) **Growth phenotypes of *S. cerevisiae* Σ1278b wild-type strain L5487 (complemented with pRS415 (*LEU2*)) carrying an empty vector pYES2 (*URA3*) or pYES2-CAN1 (*URA3*). After overnight cultivation in SC-Leu-Ura liquid medium, approximately 10^6^ cells of each strain, and serial dilutions of 10^-1^ to 10^-4^ (from left to right) were spotted and incubated onto SCGal-Leu-Ura-Lys-Arg-His agar medium in the absence (Control) and presence of excess basic amino acids (25 mM lysine (+ Lys), 25 mM arginine (+ Arg), or 5 mM histidine (+ His)). **(B)** Intracellular contents of basic amino acids in strain L5487 (complemented with pRS415) carrying an empty vector pYES2 (white) or pYES2-CAN1 (gray). The values are the means of two independent experiments that produced similar results. **(C)** Growth phenotypes of strain L5487 (complemented with pRS415) carrying an empty vector pYES2, pYES2-CTR1, pYES2-CTR3, and pYES2-FRE1. After overnight cultivation in SC-Leu-Ura liquid medium, approximately 10^6^ cells of each strain, and serial dilutions of 10^-1^ to 10^-4^ (from left to right) were spotted and incubated onto SCGal-Leu-Ura-Lys-Arg-His agar medium in the absence (Control) and presence of excess levels of basic amino acids (25 mM lysine (+ Lys) or 5 mM histidine (+ His)). **(D)** Growth phenotypes of strain L5487 (complemented with pRS415 and pRS416 (*URA3*)) under various concentrations of copper. After overnight cultivation in SD liquid medium, which contains 0.25 µM CuSO_4_, approximately 10^6^ cells of each strain, and serial dilutions of 10^-1^ to 10^-4^ (from left to right) were spotted and incubated onto SD-Cu agar medium in the presence of 0.0025, 0.025, 0.25, 2.5, or 25 µM CuSO_4_ and in the absence (Control) and presence of 5 mM histidine (+ His). **(E)** Intracellular levels of copper ions of strain L5487 (complemented with pRS415 and pRS416) in the absence (0 mM His) and presence of excess histidine (5 or 10 mM His). The values are the means and standard deviations of three independent experiments. Asterisks indicate a significant increase in copper levels compared to the control sample (0 mM His) (*p* < 0.05). **(F)** Growth phenotypes of strain L5487 (complemented with pRS415 and pRS416) (shown as ρ^+^) and its spontaneous ρ^- ^mutant. After overnight cultivation in SD liquid medium, approximately 10^6^ cells of each strain, and serial dilutions of 10^-1^ to 10^-4^ (from left to right) were spotted and incubated onto SD agar medium in the absence (Control) and presence of 5 mM histidine (+ His) under aerobic or anaerobic conditions.

To understand the mechanism by which excess of basic amino acids mediate cytotoxicity, we examined cell growth of *S. cerevisiae* under culture conditions supplemented with an elevated concentration of lysine, arginine, or histidine. As shown in Figure 1A, 5 mM of histidine severely impaired the growth of yeast cells, although a higher concentration of lysine or arginine (25 mM) did not affect growth. The concentrations for three basic amino acids were selected based on the intracellular contents of these amino acids in L5487 cells (Figure 1B). When the *CAN1* gene, encoding basic amino acids permease on the plasma membrane 12,15,38, was overexpressed, there was little effect on growth on SCGal medium that contained no excess of basic amino acids. In contrast, the overexpression of *CAN1* markedly inhibited growth under elevated levels of basic amino acids. In particular, the growth of yeast cells that overexpress *CAN1* was relatively slow on the medium in the presence of 5 mM of histidine. We confirmed that the overexpression of *CAN1* increased the intracellular levels of basic amino acids in SCGal medium (approximately 1.6- to 4.1-fold increase) (Figure 1B). Thus, these results suggest that the excess amount of intracellular basic amino acids exerts toxic effects on yeast cells. Considering that Can1 preferentially transports lysine and arginine 15, overexpression of the high-affinity histidine transporter gene *HIP1* 15,21 might enhance histidine uptake and effectively confer more severe toxicity to yeast cells in the presence of excess histidine. As histidine conferred more sensitivity to yeast cells than lysine and arginine despite having the lowest intracellular level, we further analyzed histidine cytotoxicity.

To identify multicopy suppressor genes that alleviate histidine toxicity, a yeast genomic library YEp51B 39 was introduced into L5487 cells overexpressing *CAN1*, and the transformants were screened for growth on SCGal medium containing 10 mM histidine. Sixteen independent genomic DNA fragments were isolated from the transformant colonies, and 25 full-length open reading frames were included in these fragments. After subcloning into pYES2, each gene was tested for its effect on the growth of L5487 cells in the presence of excess histidine. Consequently, the *CTR1* gene, which encodes a high-affinity copper transporter that predominantly mediates copper uptake under low copper conditions 32, exhibited the most significant suppression of the histidine-caused growth defect (Figure 1C). Although the overexpression of *CTR1* reduced cell growth in the presence of excess lysine by unknown mechanism(s), it is suggested that Ctr1 functions in alleviating histidine toxicity. Another high-affinity copper transporter gene, *CTR3* 33, and a cupric reductase gene, *FRE1*, the latter of which is required for conversion of copper(II) to copper(I) ions prior to uptake 34, also suppressed the growth defect under histidine-excess conditions when overexpressed. In addition, deletion of the *CTR1* or *FRE1* gene slightly increased sensitivity to excess histidine (data not shown). These results indicate that the histidine cytotoxicity in *S. cerevisiae* is alleviated by enhancement of copper uptake. In agreement with these results, the growth defect caused by 5 mM histidine became significantly more severe when the concentration of CuSO_4_ in SD medium was decreased (2.5 and 25 nM), although higher concentrations of CuSO_4_ (2.5 and 25 µM) suppressed the histidine toxicity (Figure 1D). It has been well studied that histidine directly binds to copper(II) to form copper(II)-bis(L-histidinato) complex under physiological conditions as in human blood 24. However, considering that the overexpression of *CAN1* enhanced histidine toxicity (Figure 1A), it seems unlikely that the elevated level of histidine simply chelates copper(II) ions outside of the cells to inhibit copper uptake. Instead, excess histidine might interact with copper(I) ions to reduce the availability of copper after incorporation into yeast cells. To verify this hypothesis, we quantified the intracellular level of copper ions. Although cell growth was delayed starting after 4-hour incubation with 5 or 10 mM histidine (data not shown), intracellular copper ions were slightly increased in the histidine-treated cells (Figure 1E). Therefore, our data consistently demonstrate that an excess level of histidine enhances copper uptake but impairs copper availability in yeast cells. Regarding the *CTR1*-overexpressing cells (Figure 1C), sufficient copper ions might be incorporated into cells in bioavailable forms and thus contributed to relieving histidine toxicity. In a similar manner as clioquinol 40, histidine may act as both a chelator and an ionophore: histidine chelates copper(II) outside of the cell and is taken in as a complex, which may facilitate uptake of copper ions, though copper ions in this complex are not bioavailable. Additionally, it is possible that histidine-induced copper deficiency upregulates expression of copper transporters, which may increase copper uptake.

What functions of copper does excess histidine inhibit? Intracellular copper is distributed to distinct target proteins via specific cytosolic copper chaperons, such as Atx1, Ccs1, and Cox17. Atx1 assists in the transport of copper to the cell-surface iron uptake protein Fet3 through the function of Ccc2, which has copper-transporting ATPase activity, on the post-Golgi vesicle 41. Ccs1 delivers copper specifically to the superoxide dismutase Sod1, which scavenges reactive oxygen species in the intermembrane space of mitochondria 42,43. Another copper chaperon Cox17 transfers copper to the mitochondrial inner membrane proteins Cox11 and Sco1, both of which are essential for assembly of cytochrome *c* oxidase, which is the last enzyme in the respiratory electron transport chain 44. In this study, we tested whether histidine cytotoxicity might be mediated by defective mitochondrial functions due to reduced copper availability. As shown in Figure 1F, L5487 cells were clearly sensitive to 5 mM histidine on SD plates, although histidine cytotoxicity was completely abrogated by a spontaneous cytoplasmic *petite* (ρ^-^) mutation, which inactivates mitochondrial respiratory functions. It is also worth noting that yeast cells showed similar growth phenotypes in the absence or presence of excess histidine under anaerobic conditions (Figure 1F). Thus, histidine cytotoxicity was observed only when mitochondrial aerobic respiration should be functional. We hypothesize that the deficiency of the Cox17-bound copper ions due to excess of intracellular histidine might cause the abnormal assembly of cytochrome *c *oxidase complex in aerobically growing cells, leading to the observed toxic effect. This might be supported by the fact that the disturbance of cytochrome *c* oxidase induces apoptosis-like cell death in *S. cerevisiae* 45. However, we cannot rule out the possibility that defective mitochondrial respiration reduces incorporation of histidine by some unknown mechanism(s), and hence, the excess level of histidine did not elicit the toxicity in ρ^-^ cells under aerobic conditions or in ρ^+^ cells under anaerobic conditions.

In this study, we discovered the cytotoxicity of excess histidine in *S. cerevisiae*, which is tightly associated with the reduced availability of intracellular copper ions. Similarly, Pearce and Sherman 37 revealed that intracellular histidine synthesis is required for the detoxification of excess copper. Both studies commonly suggest that intracellular histidine has a novel and important role in copper homeostasis.

## MATERIALS AND METHODS

### Strains and Plasmids

The *S. cerevisiae* strain used in this study was Σ1278b wild-type strain L5487 (*MATα ura3-52 leu2::hisG*), which was generously provided by Gerald Fink (Whitehead Institute). A spontaneous cytoplasmic *petite* (ρ^-^) mutant of L5487 was isolated, according to Fox *et al.* 46. *Escherichia coli* strain DH5α (F^-^λ^-^Φ*80lacZ*∆*M15* ∆(*lacZYA argF*)*U169 deoR recA1 endA1 hsdR17*(*r_k_*^-^*m_k_*^+^) *supE44 thi-1 gyrA96*) was used to subclone the yeast gene and construct plasmids. Low-copy plasmids pRS415 and pRS416 24 were used to complement auxotrophic mutations *ura3* and *leu2*, respectively. A galactose-inducible plasmid pYES2 (Life Technologies) was used for overexpression of the *CAN1*, *CTR1*, *CTR3*, and *FRE1* gene. A 2 µm-based yeast genomic library YEp51B 12 was used to identify multicopy suppressor genes of *CAN1-*overexpressing L5487 strain.

### Culture Media

The media used for growth of *S. cerevisiae* were a synthetic complete medium, SC (2% glucose, 0.67% yeast nitrogen base without amino acids (Difco), supplemented with synthetic drop-out amino acid and nucleotide mixture as required), and a synthetic defined medium, SD (2% glucose, 0.67% yeast nitrogen base without amino acids (Difco)). For overexpression of *CAN1*, *CTR1*, *CTR3*, and *FRE1*, SC with galactose as a carbon source (SCGal) (2% galactose, 0.67% yeast nitrogen base without amino acids (Difco), supplemented with synthetic drop-out amino acid and nucleotide mixture as required) was used. To evaluate the effect of copper sulfate (CuSO_4_) addition, SD-Cu media (2% glucose, 0.67% yeast nitrogen base without amino acids and copper (ForMedium)) containing different concentrations of CuSO_4_ were used. All experiments were performed at 30°C, and all growth media were adjusted to pH 6.5 with HEPES buffer (pH 7.0) and sodium hydroxide. When necessary, 2% agar was added to solidify the medium. For anaerobic cultivation, the inoculated plates were incubated with O_2_-absorber/CO_2_-generator AnaeroPouch-Anaero (Mitsubishi Gas Chemical Company) and O_2_ indicators. The *E. coli* recombinant strains were grown in Luria-Bertani complete medium containing 50 µg/ml ampicillin or M9 minimal medium plus 2% Casamino acids containing 50 µg/ml ampicillin. If necessary, 2% agar was added to solidify the medium.

### Measurements of Intracellular Amino Acids Levels

According to a method described previously 3, intracellular amino acids were extracted by boiling from log-phase cells cultivated in SCGal-Leu-Ura liquid medium, and were subsequently quantified with an amino acid analyzer AminoTac JLC-500/V (JEOL).

### Determination of Intracellular Copper Ions

Cell lysates were prepared from log-phase cells cultivated in SC-Leu-Ura-Lys-Arg-His liquid medium, and copper ions concentrations were determined by the CUPRAC-BCS assay 25.
